# Neoadjuvant use of methotrexate in eosinophilic angiocentric fibrosis of upper lip and hard palate: A case report

**DOI:** 10.1111/dth.15094

**Published:** 2021-08-13

**Authors:** Francesca Nastro, Angelo Ruggiero, Gustavo Spanò, Sara Cacciapuoti, Mariateresa Cantelli, Gabriella Fabbrocini, Claudio Marasca

**Affiliations:** ^1^ Section of Dermatology, Department of Clinical Medicine and Surgery University of Naples Federico II Napoli Italy

## CONFLICT OF INTEREST

The authors have declared no conflicting interests.

## ETHICS STATEMENT

All procedures adopted in the present study were in respect to the ethical standards in the World Medical Association Declaration of Helsinki. The subject gave his written informed consent to publish the present case (including publication of the image).

Dear Editor,

Eosinophilic angiocentric fibrosis (EAF) is a rare, idiopathic fibroinflammatory disease usually involving upper airways, nasal cavity, paranasal sinuses, and rarely the orbit. The most common presentation is a nasal mass with prolonged obstructive symptoms.^1^, ^2^ Mucosal ulceration and bone destruction may be observed.^3^, ^4^, ^5^ Diagnosis is made through histological examination and it may show small vessel eosinophilic‐vasculitis with adjacent inflammatory infiltrate of lymphocytes and plasma‐cells, or an obliterative concentric perivascular fibrosis in an “onion skin” pattern as in the later fibrotic stage.^6^ Several medical treatments have been proposed including immunosuppressant‐drugs and systemic steroids, however, surgical approach still represents the only effective treatment.^7^ Chronic inflammation may lead to the development of an immunocompromised district, which is a skin area more vulnerable than the rest of the body for genetic or acquired reasons, prone to developing opportunistic infections, tumors, or dysimmune reactions.^8^

**FIGURE 1:**
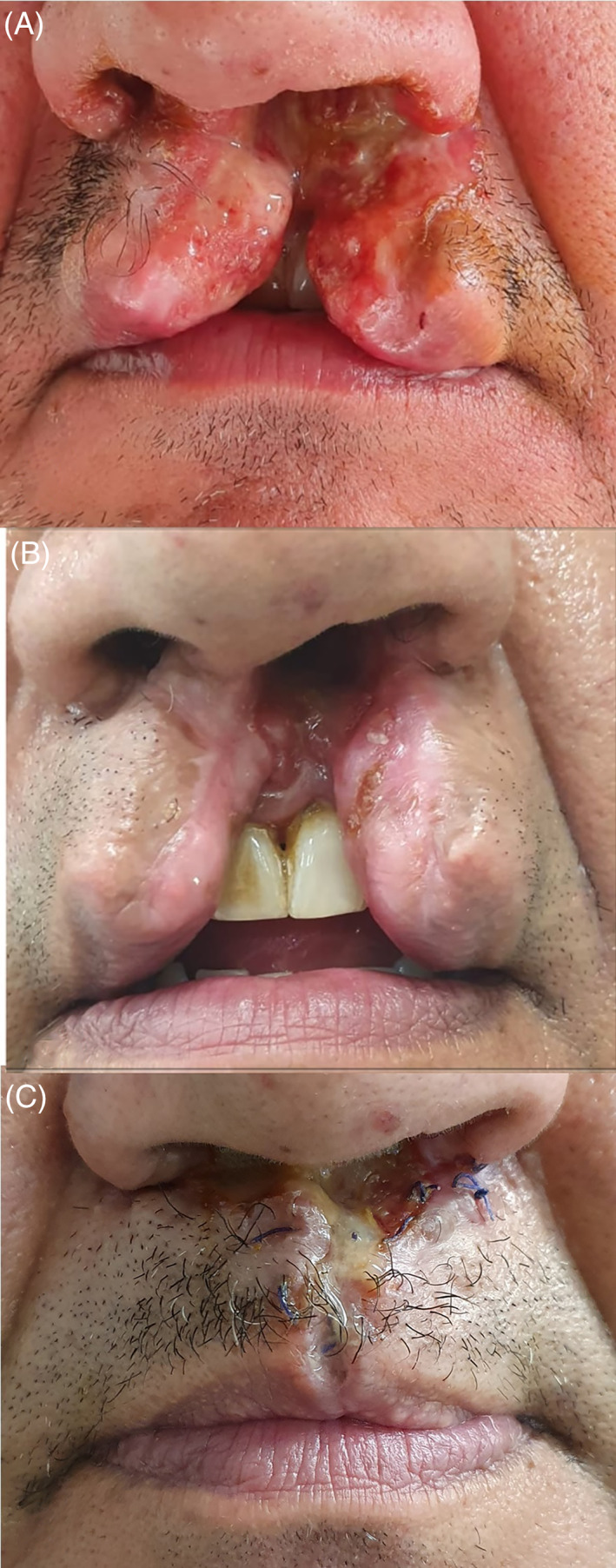
Upper lip and nasal lesions of eosinophilic angiocentric fibrosis at baseline (A), after 3 weeks of medical treatment (methotrexate 12.5 mg/week + predisolone 50 mg/die −0.5 mg/kg body weight) (B), and after surgery (C)

Herein we report the case of a 46‐year‐old man showing a destructive lesion of upper lip, nasal cavity, and cleft palate. His medical history was positive for chronic cocaine abuse and plaque psoriasis not controlled by topical treatment. At the first examination, the patient showed an exuding‐destructive lesion involving upper‐lip, the nasal‐choanae and hard‐palate, with local edema and speech impairment (Figure 1A). Patient underwent incisional biopsy of the lesion. Histopathological examination revealed angiocentric fibrosis with onion skin pattern and perivascular exudates of eosinophils accompanied by plasma cells and lymphocytes. There was absence of fibrinoid necrosis. These findings were diagnostic for EAF. Laboratory investigations revealed raised erythrocyte sedimentation rate and C‐reactive protein, however complete blood count was in the normal range. Autoimmune screening, including antinuclear antibody, antineutrophil cytoplasmic antibody (ANCA) and rheumatoid factor, was negative. Oral prednisolone 75 mg (0.75 mg/kg body weight) was started, without showing any clinical improvements after 1 month‐therapy. Therefore, considering patient's medical history of plaque psoriasis, steroid induced worsening of the chronic hypertension, and methotrexate anti‐inflammatory properties related to the effects on adenosine,^9^ the association with subcutaneous methotrexate and prednisone was started (methotrexate 12.5 mg/week + prednisone 50 mg/die −0.5 mg/kg body weight). After 3 weeks of treatment, a reduction of local edema and exudation was observed (Figure 1B), and at week 6 patient also showed the absence of cleft hard palate, referring a speech improvement as well. Steroid treatment was reduced over the next 6 weeks while methotrexate was maintained at the same dosage, maintaining clinical outcomes. Patient underwent surgical reconstruction of upper lip and hard palate with excellent functional and aesthetic results (Figure 1C). Methotrexate treatment was suspended a month after the surgical reconstruction. No new clinical manifestations and/or burden of the diseases were observed at last follow‐up visit (10 months).

Although in literature several cases of EAF have been described,^1^, ^2^, ^3^ our case is atypical for several reasons. First, there is a simultaneous involvement of the nasal cavity, hard palate and upper lip. Indeed, while few reported cases described an involvement of sinonasal tract,^4^, ^5^ upper airways and orbits, no cases of lip or hard palate localization have been reported. Furthermore, a beneficial effect of methotrexate on wound healing was observed in our EAF patient. To date no guidelines have been reported on the EAF‐treatment and case reports or large studies on effectiveness of methotrexate in EAF patients are still lacking, even if other immunomodulant‐agents (dapsone, azathioprine, and hydroxicloroquine) have been tried. Finally, in this article we describe a case of EAF in a patient with a history of chronic cocaine abuse, assuming a possible association between EAF and chronic cocaine abuse. However, further study is needed to confirm our results and better clarify the possible role of methotrexate as neoadjuvant treatment in EAF in order to improve surgical outcomes.

## Data Availability

The data that support the findings of this study are available from the corresponding author upon reasonable request.
